# Amputation Versus Limb-Salvage Surgery as Treatments for Pediatric Bone Sarcoma: A Comparative Study of Survival, Function, and Quality of Life

**DOI:** 10.7759/cureus.78543

**Published:** 2025-02-05

**Authors:** Sara S Vale, Rui Castro, Alexandra Andrade, Joana Faleiro, Nina Abreu, Cristina Mendes, Jean-Pierre Gonçalves

**Affiliations:** 1 Pediatrics, Unidade Local de Saúde Região de Leiria Entidade Pública Empresarial (EPE), Leiria, PRT; 2 Family Medicine, Unidade Local de Saúde Médio Ave Entidade Pública Empresarial (EPE), Santo Tirso, PRT; 3 Pediatrics, Hospital Central do Funchal, Serviço de Saúde da Região Autónoma da Madeira (SESARAM) Estabelecimento Público Empresarial da Região Autónoma da Madeira (EPERAM), Funchal, PRT; 4 Pediatrics, Instituto Português de Oncologia de Lisboa Francisco Gentil Entidade Pública Empresarial (EPE), Lisbon, PRT

**Keywords:** amputation, ewing sarcoma, limb-salvage surgery, malignant bone cancer, osteosarcoma, osteosarcoma research, pediatric bone tumor, pediatric oncology, quality of life (qol)

## Abstract

Background: Pediatric appendicular bone sarcomas, including osteosarcoma and Ewing's sarcoma, are rare but aggressive malignancies that have a profound impact on survival, physical function, and quality of life (QoL). Treatment options usually involve either limb-salvage surgery (LSS) or amputation (AMP), although evidence about outcomes among these approaches is still limited.

Methods: A retrospective cohort study was conducted involving pediatric cases treated for appendicular bone sarcomas between 2000 and 2021 at the Instituto Português de Oncologia de Lisboa (IPOL), a Portuguese oncology center in Lisbon. It evaluated functional and QoL outcomes and survival. Patients were stratified by surgical approach (LSS vs. AMP) and evaluated by validated outcome tools, including the Toronto Extremity Salvage Score (TESS) and the Medical Outcomes Study Short-Form 36 version 2 (MOS SF-36v2) QoL questionnaire. P-values <0.05 were considered significant.

Results: A total of 62 patients were included, with an overall five-year survival rate of 38%. Poorer survival outcomes were recorded among those presenting with metastatic disease, larger tumor sizes (>8 cm), and those who underwent AMP. Limb-salvage surgery was performed in 59.7% of cases and presented higher mean functional scores (88.4%) compared with AMP (79%). The QoL scores varied, with LSS patients reporting better outcomes in physical and emotional domains. Patients from Portuguese-speaking African countries (PSAC) had poorer survival rates, a finding that reflects disparities that could be related to advanced disease presentation and limited health resources.

Conclusion: Limb-salvage surgery had better functional outcomes and QoL compared to AMP. However, survival of these patients remains a challenge, especially for those from resource-limited settings. These results highlight the need for early diagnosis, improvement in access to healthcare, and further research to improve treatment.

## Introduction

Malignant bone tumors have an estimated annual incidence of seven cases per 1,000,000 children under the age of 16, representing 4.7% (ages 0-14) and 7.8% (ages 15-19) of all pediatric cancers [1]. Within this age group, osteosarcoma is the most frequently diagnosed primary malignant bone tumor, followed by Ewing’s sarcoma [2-5]. Evidence on the geographic and ethnic distribution of these diseases is conflicting [5-8], although certain countries appear to report higher yearly case numbers. Portugal, for instance, has an age-standardized incidence rate (ASR) for osteosarcoma of 5.1 cases per million person-year [5], slightly above the global average of three to five per million [9-10]. Potential explanations include variations in genetic predisposition, lifestyle, environmental exposures, healthcare access, diagnostic capabilities, reporting practices, and even socioeconomic factors or uneven research investment [6]. Earlier detection through stronger screening programs and increased awareness can also lead to higher recorded rates.

European data point to five-year survival rates of around 50% for osteosarcoma and 52% for Ewing's sarcoma, which have risen to about 60% over the last decade. These improvements reflect advances in surgery, chemotherapy, multidisciplinary strategies, and the broader adoption of standardized treatment protocols [10]. The presence of metastatic disease at diagnosis is a key determinant of survival [11] since the five-year survival rates for patients with local disease, 80% for osteosarcoma and 65% to 70% for Ewing’s sarcoma [12], are significantly higher than the 20% to 30% and the 25% to 35% reported, respectively, for osteosarcoma and Ewing’s sarcoma with metastatic disease at presentation.

The standard of care for bone sarcomas consists of neoadjuvant chemotherapy, complete surgical resection of the primary tumor, and/or radiotherapy, followed by adjuvant chemotherapy [13-14]. Different drug combinations and protocols have been proposed depending on the specific diagnosis and tumor grade [15-23]. Securing complete local control remains essential for a cure.

A decision is then made between two main surgical approaches, namely, amputation (AMP) or limb-salvage surgery (LSS). Factors influencing this decision include the equivalent likelihood of cure (based on radiological evaluation), risk of complications, and acceptable functional and cosmetic outcomes for the child and parents. Most children with osteosarcoma are currently eligible for LSS, with several reconstruction methods available [24]. The current literature is conflicting on whether survival rates are the same for both surgical approaches [25-27], or if LSS yields a significant survival advantage [27-30]. Nevertheless, LSS appears to consistently preserve more function [25, 2, 28, 31, 32] and achieve higher scores in certain quality of life (QoL) domains, even though its overall performance sometimes mirrors that of AMP [32-35]. Moreover, the surgical level (i.e., where the procedure is performed anatomically) appears to influence functional and personal satisfaction outcomes [31-32]. 

Malignant bone tumors most commonly affect the lower limb, particularly around the knee (distal femur and proximal tibia) [5,23]. Given this typical location, the essential role of surgery in treatment, and the age group most frequently affected, these tumors can pose a substantial burden with a marked impact on function and QoL.

Because there is limited empirical evidence for our specific study population, we aim to assess the efficacy of AMP and LLS in children and adolescents treated surgically for appendicular osteosarcoma or Ewing’s sarcoma, focusing on patient survival, functional preservation, and quality of life.

## Materials and methods

The target population comprised patients treated for appendicular bone sarcoma (osteosarcoma and Ewing’s sarcoma) at the Instituto Português de Oncologia de Lisboa (IPOL), a Portuguese oncology center in Lisbon, between March 2000 and March 2021, who were under 16 years at hospital admission. We excluded patients lost to follow-up, those who underwent surgery in other centers, and those diagnosed with non-appendicular disease.

We developed a retrospective, analytical, observational cohort study. Data were collected by reviewing the enrolled participants’ medical records, and all relevant information for each variable was grouped according to the patient’s diagnosis and surgery type. Functional and QoL assessments were conducted using peer-reviewed, questionnaire-based measurement scales.

Follow-up time-based survival functions were generated to estimate the five-year survival rates of the overall study sample and to compare five-year survival across patient nationality (Portuguese vs. Portuguese-speaking African countries (PSAC)), diagnosis, primary tumor size (using an 8 cm cut-off due to its prognostic significance [2]), presence of metastatic at staging, time of intervention, and surgery approach.

Various measures exist for functional assessment in different medical fields. One widely used, peer-reviewed measure is the Toronto Extremity Salvage Score (TESS), specifically designed to evaluate preserved function in individuals with upper or lower limb bone or soft tissue sarcomas following curative surgery. The TESS scores range from 0 to 100, with higher scores indicating better functional preservation [36]. One of the main strengths of TESS is its patient-reported design, which provides direct insight into a person's self-perceived disability. For the QoL assessment, we used the Medical Outcomes Study Short-Form 36 version 2 (MOS SF-36v2) questionnaire [37]. Each dimension of this questionnaire also yields a 0 to 100 score, with higher values signifying better-perceived health status or quality of life.

Eligible participants for functional and quality of life assessments were contacted by phone, and, after obtaining verbal permission, the TESS and MOS SF-36v2 questionnaires were mailed to them along with the informed consent documents. Scoring for each measure involved calculating the patient's reported score relative to the total possible score. For the TESS, we compiled the final score from either the upper or lower limb version of the questionnaire, then separately scored the two global self-perception questions regarding disability and difficulty in daily living. For the MOS SF-36v2, each of the eight questionnaire dimensions was scored independently. The resulting scores were then compared between the two surgery groups.

A translated and cross-culturally validated equivalent of the MOS SF-36v2 questionnaire was used with the authors' permission [38-40]. As no equivalent version of the TESS was available at the time, we conducted a translation and cross-cultural adaptation of the original TESS measure after securing the original author's authorization, following recommended guidelines [41]. 

All descriptive and inferential statistical analyses were carried out using the IBM SPSS® Statistics software, version 26.0 (IBM Corp., Armonk, NY). Statistical significance was set at p<0.05, with a 95% confidence interval.

No data or variables enabling patient identification were stored or included in the final paper. To ensure confidentiality, each participant was assigned a coded identifier in place of their name and process number; the link between the code and the patient was kept in a password-encrypted digital file. This study was approved by the Ethics Committee for Life and Health Sciences Research (CEICVS) under protocol number CEICVS 024/2022. According to the Portuguese National Ethics Committee for Clinical Research (CEIC) guidelines [42], participants aged 16 or older gave their informed consent independently, while those under 16 years of age (but over 14 years) provided informed assent and were represented by their legal guardians. However, if a participant chose not to enroll, their decision took precedence irrespective of age. To ensure no cost to participants, mailing of the questionnaires included return postage.

## Results

A total of 62 participants were enrolled in the study. After excluding the deceased patients, 23 were eligible for functional and QoL assessment. Of these, five were excluded due to either not completing the MOS SF-36v2 or having an invalid TESS score, leaving 18 participants for the final evaluation. Osteosarcoma was the most common diagnosis (n=50; 80.6%), and most patients were submitted to LSS (n=37; 59.7%) rather than curative AMP (n=14; 22.6%). Table 1 and Table 2 present the population characteristics by diagnosis and surgery type, respectively.

**Table 1:** Patient characteristics according to tumor diagnosis (2000-2021) Min: minimum; Max: maximum; Yrs: years; PSAC: Portuguese-speaking African countries; QoL: quality of life. P-values correspond to comparisons between Osteosarcomas and Ewing sarcoma groups. Statistical significance is indicated as follows: p < 0.05 (*)

Characteristics		All patients n (%)	Osteosarcoma n(%)	Ewing's sarcoma n(%)	p-value
Sex	Male	31 (50.0)	24 (48.0)	7 (58.3)	0.520
	Female	31 (50.0)	26 (52.0)	5 (41.7)	
Origin	Portuguese	46 (74.2)	34 (68.0)	12 (100)	0.023*
	PSAC	16 (25.8)	16 (32.0)	0	
Age at diagnosis (yrs)	Median	12	13	10	0.034*
	Min-Max	4-16	4-17	4-14	
Age at functional/QoL assessment (yrs)	Median	19	18	20	0.285
	Min-Max	11-28	11-28	20-26	
Time of diagnosis	2000-2010	27 (43.5)	20 (40.0)	7 (58.3)	0.250
	2011-2021	35 (56.5)	30 (60.0)	5 (41.7)	
Limb	Inferior	53 (85.5)	44 (88.0)	9 (75.0)	0.251
	Superior	9 (14.5)	6 (12.0)	3 (25.0)	
Tumor size (cm)	≤8	16 (30.8)	13 (30.2)	3 (33.3)	0.571
	>8	36 (69.2)	30 (69.8)	6 (66.7)	
Bone sarcoma	Ewing	12 (19.4)	-	-	-
	Osteosarcoma	50 (80.6)	-	-	-
Local treatment	Amputation	14 (22.6)	12 (28.6)	2 (22.2)	0.699
	Limb-salvage	37 (59.7)	30 (71.4)	7 (77.8)	
Metastatic disease		23 (37.1)	19 (38.0)	4 (33.3)	0.764
Time of follow-up (yrs)	Median	3	3	4	-
	Min-Max	0-19	0-19	1-16	0.750
Death event		39 (62.9)	31 (62.0)	8 (66.7)	0.764

**Table 2:** Patient characteristics according to surgery type (2000-2021) Min: minimum; Max: maximum; Yrs: years; PSAC: Portuguese-speaking African countries; QoL: quality of life. P-values correspond to comparisons between limb-salvage surgery and amputation groups. Statistical significance is indicated as follows: p < 0.05 (*)

Characteristics		Amputation n (%)	Limb-salvage n (%)	p-value
Sex	Male	11 (78.6)	15 (40.5)	0.027*
	Female*	3 (21.4)	22 (59.5)	
Origin	Portuguese	7 (50.0)	34 (91.9)	0.002*
	PSAC	7 (50.0)	3 (8.1)	
Age at diagnosis (yrs)	Median	10	14	0.138
	Min-Max	4-15	5-16	
Age at functional/QoL assessment (yrs)	Median	14	19	0.335
	Min-Max	-	11-28	
Time of diagnosis	2000-2010	7 (50.0)	16 (43.2)	0.452
	2011-2021	7 (50.0)	21 (56.8)	
Limb	Lower	14 (100)	29 (78.4)	0.061
	Upper	-	8 (21.6)	
Tumor size (cm)	≤8	4 (33.3)	11 (37.9)	0.536
	>8	8 (66.7)	18 (62.1)	
Bone sarcoma	Osteosarcoma	12 (85.7)	30 (81.1)	0.527
	Ewing Sarcoma	2 (14.3)	7 (18.9)	
Metastatic disease		5 (35.7)	8 (21.6)	0.247
Time of follow-up (yrs)	Median	10	5	-
	Min-Max	5-16	1-16	0.214
Death event		12 (85.7)	17 (45.9)	0.010*

The study sample was evenly split by sex (31 males and 31 females). Of those, 46 (74.2%) were Portuguese, and 16 (25.8%) were from PSAC countries. The median age at diagnosis was 12 years (four to 16), and the median age at functional/QoL assessment was 19 years (11 to 28). In most cases, the primary tumor was located in the lower limb (n=53, 85.5%), while only nine (14.5%) presented upper limb involvement. Tumor size information was missing for 10, but among the 52 who had available data, most cases extended over eight centimeters along their largest axis (n=36; 69.2%). The median follow-up period was three years (ranging from 0 to 19 years). At diagnosis, 23 patients (37.1%) already had metastatic disease. During the follow-up period, 39 deaths (62.9%) were reported, of which 12 (30.8%) occurred among patients from PSAC.

Diagnosis and treatment

Among the 50 patients diagnosed with osteosarcoma, 46 received neoadjuvant chemotherapy, with 41 adhering to the European and American Osteosarcoma Study (EURAMOS), which includes high-dose methotrexate, doxorubicin, and cisplatin. Of the 41 patients who underwent surgical resection, the extent of tumor necrosis was categorized as follows: less than 50% in 10 patients (poor response), between 50% and 90% in 18 patients (moderate response), between 90% and 99% in 10 patients (good response), and 100% in three patients (excellent response).

Conversely, all 12 patients diagnosed with Ewing’s sarcoma received neoadjuvant chemotherapy, with 11 following the Euro Ewing99 protocol, which consists of alternating cycles of vincristine, doxorubicin, and cyclophosphamide (VDC) with ifosfamide and etoposide (IE). Among these, nine underwent surgical intervention. Of the surgically treated Ewing’s sarcoma patients, six demonstrated a good pathologic response (tumor necrosis >90%), while three exhibited a poor response (tumor necrosis <90%).

A total of 51 patients received curative surgical treatments, all performed by orthopedic surgeons. Of these, 14 patients (22.6%) underwent AMP (12 with osteosarcoma and two with Ewing's sarcoma), while 37 patients (59.7%) underwent LSS (30 with osteosarcoma and seven with Ewing's sarcoma). Within the LSS group, 11 patients (29.7%) required multiple surgical procedures. Additionally, 11 patients (17.7%) presented with advanced, inoperable disease or underwent palliative amputation or disarticulation.

Significant heterogeneity

Significant heterogeneity emerged in terms of nationality and diagnosis. All 16 patients of PSAC-origin patients treated at the Instituto Português de Oncologia de Lisboa Francisco Gentil (IPO-LFG) had osteosarcoma, with none presenting Ewing’s sarcoma. Patients diagnosed with Ewing's sarcoma had a significantly lower median age of diagnosis (10 years) compared to those with osteosarcoma (13 years). No other category showed marked differences.

When analyzing sex and place of origin, the AMP group comprised mostly males (11/14, 78.6%), whereas the LSS was predominantly female (22/37, 59.5%). The LSS group also consisted mainly of Portuguese-origin children (34/37; 91.9%) with only three from PSAC (3/37, 8.1%). Unlike the grouping by diagnosis, significant differences were found in mortality between the surgical groups (AMP 85.7% vs. LSS 45.9%, p=0.010). No other notable differences emerged.

**Figure 1:**
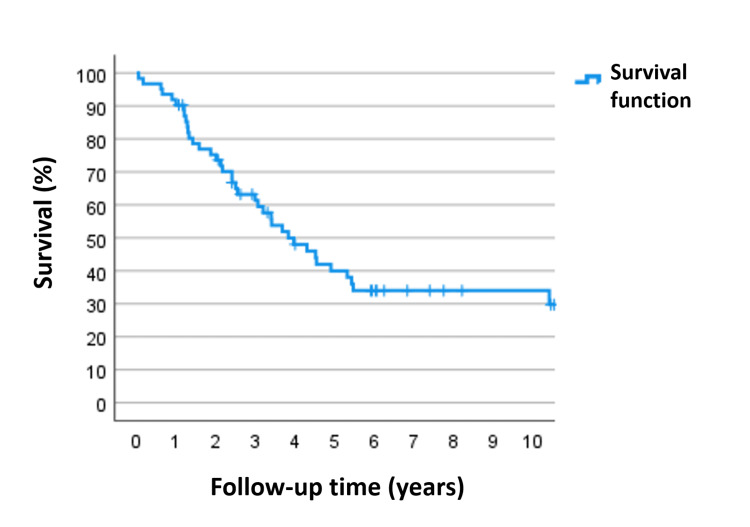
Overall survival function of the study sample

Overall, the five-year survival rate for the entire study sample was roughly 38%, which is lower than the published figures referenced earlier. As shown in Figure 1, the event count stabilizes after the fifth year of follow-up and remains unchanged until around the tenth year.

We then organized the participants by their various characteristics to explore differences in survival, aiming to identify potential associations between these characteristics and reduced survival.

**Figure 2:**
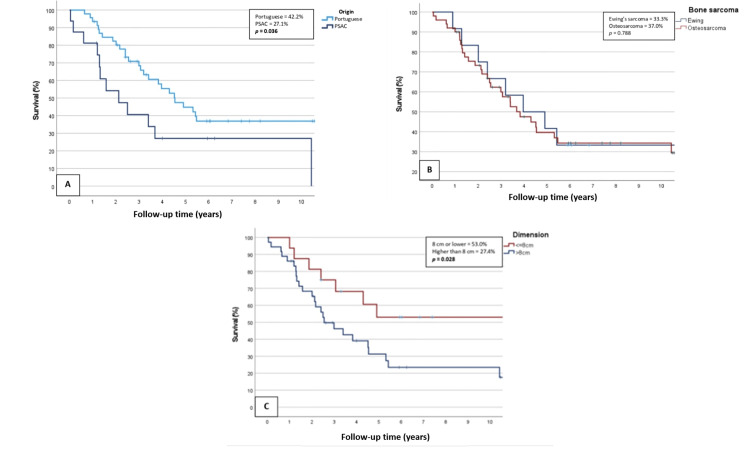
Survival curves of the patients when grouped by variables A) By place of origin; B) By type of tumor; C) By tumor dimension Percentage values indicate the five-year survival rates.

**Figure 3:**
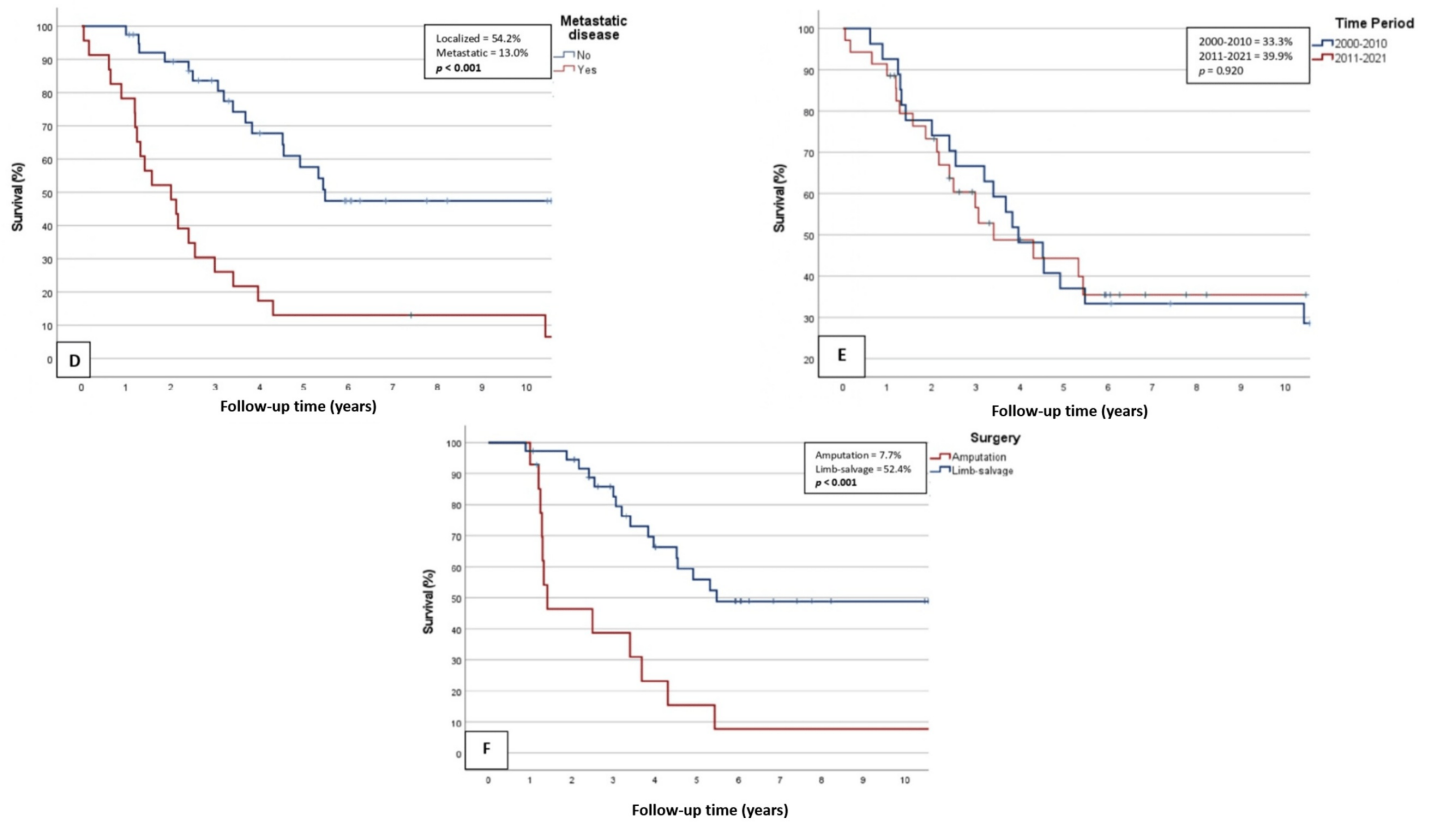
Survival curves of the patients when grouped by variables D) By the presence of metastatic disease; E) By the time period of intervention; F) By the type of surgical procedure applied. Percentage values indicate the five-year survival rates.

Figures 2-3 present comparative survival graphs for participants grouped according to those characteristics.

A statistically significant decrease in the five-year patient survival rates was observed in patients with PSAC origin (27.1% vs. 42.2% for Portuguese origin; p=0.036), tumor sizes exceeding 8 centimeters (27.4% vs. 53%, p=0.028), metastatic disease at initial staging (13% vs. 54.2% for localized disease, p<0.001), and AMP procedures (7.7% vs. 52.4% for LSS, p<0.001). No significant differences emerged based on the sarcoma subtype or treatment period.

To further explore the predictors of mortality suggested by the Kaplan-Meier curves, a Cox proportional hazards model was applied, adjusted for the variables analyzed in survival. The final results are summarized in Table 3.

**Table 3:** Adjusted cox proportional hazards model for mortality The initial Cox regression model adjusted for gender, origin, age at diagnosis, bone sarcoma type, and treatment period. The final model was obtained by the backward stepwise selection method. P<0.05 was considered statistically significant.

Characteristics		Hazard ratio (95%CI)	p-value
Local treatment	Limb-salvage	1	0.001
	Amputation	3.57 (1.67-7.65)	
Metastatic disease	No	1	0.003
	Yes	3.12 (1.46-6.68)	

Using the backward stepwise method, metastatic disease (hazard ratio (HR) = 3.12, p = 0.003) and AMP (HR = 3.57, p = 0.001) were identified as the strongest risk factors, each conferring a more than threefold increase in mortality compared to localized disease and LSS, respectively.

Table 4 summarizes the performance of the elected participants on the domains of function and quality of life, represented by the mean (± standard deviation (SD)) scores of each surgery group.

**Table 4:** Function and quality of life assessment MOS SF-36v2: Medical Outcomes Study Short-Form 36 version 2; TESS: Toronto Extremity Salvage Score; Mean±SD values are shown in the limb-salvage group. P<0.05 was considered statistically significant.

Score		Amputation	Limb-salvage	p-value
TESS score (%)		79	88.4±10.3	0.390
MOS SF-36v2 dimensions (%)	Physical performance	100	77.8±42.8	0.620
	Physical functioning	55.0	63.3±30.0	0.789
	Emotional performance	100	87.0±32.6	0.704
	Mental well-being	72.0	63.6±18.7	0.667
	Vitality	60.0	57.5±20.6	0.907
	Social functioning	100	75.0±32.1	0.459
	Bodily pain	100	74.9±32.7	0.416
	General health	55.0	55.0±29.8	0.999

The TESS scores represent the patient-reported degree of preserved function, while the MOS SF-36v2 scores demonstrate the patient-reported levels of QoL, discriminated along the different domains of the questionnaire. As previously stated, 18 participants were evaluated, of whom 17 belonged to the LSS group, leaving only one patient in the curative AMP group. This large asymmetry in group sizes makes statistical analysis of differences rather challenging and limits the interpretation of significance. However, for consistency, the p-values are still presented. These results primarily allow for descriptive analysis, especially for the LSS group. The TESS scores are based on the participants' experiences during the past week, while the MOS SF-36v2 scores refer to the past four weeks.

The TESS scores

The LSS group demonstrated a higher average TESS score compared to the single AMP patient (88.4±10.3% vs. 79%). The TESS questionnaire concludes with two self-perception questions about disability and difficulty in everyday life. The AMP participant reported feeling “mildly disabled” and experiencing “a little bit of difficulty” in daily life. Among the limb-salvage group, self-perceptions varied: four participants reported being “not at all disabled,” five as “mildly disabled,” another five as “moderately disabled,” and two as “severely disabled.” Regarding everyday life difficulty, seven reported “no difficulty,” five reported “a little bit of difficulty,” and four reported “moderate difficulty.” One participant did not respond to these self-perception questions.

The MOS SF-36v2 scores

The AMP patient reported perfect scores across four QoL domains: physical performance, emotional performance, social functioning, and bodily pain. The lowest scores for this participant were observed in the general health and physical functioning domains (55%). In the limb-salvage group, the highest average scores were recorded in the emotional performance domain (87.0±32.6%), followed by the physical performance (77.8±42.8%). Similar to the AMP participant, the limb-salvage group’s lowest average score was in the general health domain (55.0±29.8%). Notably, the high standard deviation values in the MOS SF-36v2 scores for the limb-salvage group suggest considerable variability in reported QoL, a pattern not observed with the TESS scores.

This descriptive analysis highlights differences in reported function and QoL between the two groups while emphasizing the variability within the limb-salvage cohort. However, the limited representation in the AMP group precludes definitive conclusions about comparative outcomes.

## Discussion

Bone sarcomas, despite being one of the most common oncologic diagnoses in children and adolescents, remain rare in absolute numbers, as highlighted by the epidemiologic data presented earlier. This rarity makes it rather challenging to gather sufficient medical data from eligible participants to conduct robust research. Even with a relatively large time frame of 21 years, we were only able to collect data on 62 patients. While this is a reasonable sample size for the purpose, larger cohorts would enhance the validity of comparisons and align with methodologies in studies. Additionally, the high mortality associated with bone sarcomas further reduces the sample size, particularly for functional and QoL assessment.

Our cohort does not fully reflect the broader European reality of pediatric bone sarcomas, largely due to the inclusion of children from PSAC. While this inclusion introduces differences from other European and North American studies, it accurately represents the caseload of our center (IPO-LFG) and underscores the importance of globally inclusive research to characterize pediatric bone sarcomas better.

In line with the literature, no significant sex differences were observed, osteosarcoma was the most common diagnosis, and the lower limb was the most affected site. Interestingly, no PSAC-origin patients presented with Ewing’s sarcoma, which we hypothesize might be due to the tumor's propensity for rapid progression and soft tissue involvement. Combined with limited access to healthcare, this may lead to disease progression and death before referral.

Surgical treatment revealed disparities. Children of PSAC origin were more often subjected to amputation, while Portuguese-origin children were more likely to undergo limb-salvage surgery. This disparity likely reflects the more advanced disease stages observed in PSAC-origin children, which limits the feasibility of functional limb preservation.

When analyzing survival outcomes by origin, the Portuguese-only subgroup demonstrated a five-year survival rate of 42%, aligning more closely with the figures reported in European studies. This is likely reflective of earlier diagnoses and better access to advanced medical care in Portugal, which parallels the conditions in other European countries. In contrast, the PSAC-origin subgroup exhibited a significantly lower survival rate of 27%, which heavily influenced the overall cohort’s survival rate of 38%. A recent meta-analysis supports the influence of geographic variations in care standards and treatment availability on survival outcomes [1].

The strongest predictors of decreased survival were the presence of metastatic disease and the need for amputation. While metastatic disease is a well-established poor prognostic factor, the association between amputation and higher mortality reflects the more advanced disease stages requiring this intervention rather than the procedure itself [43, 44].

Additionally, PSAC-origin patients and those with tumors larger than 8 cm had lower survival, consistent with established cancer staging guidelines [2]. Tumor type and the treatment timeframe showed no significant impact, aligning with the minimal advancements in treatment over the past decade.

Due to the asymmetry in group sizes, meaningful statistical comparisons between amputation and limb-salvage groups were not feasible. Nevertheless, the limb-salvage group’s TESS scores were high and showed limited variability, consistent with other studies. These scores also exceeded those reported by the sole amputee patient. In terms of quality of life, greater variability was observed both within and across domains of the MOS SF-36v2, reflecting findings from other studies where differences are most pronounced in specific domains rather than overall scores.

The asymmetry in the composition of both groups under evaluation makes it impossible to assess the differences in performance directly and validly for amputation and limb-salvage patients. However, particularly for the limb-salvage group, we can still use descriptive statistics for direct comparison with the results reported by other studies.

On average, the limb-salvage group presented high scores on the TESS measure with little variation between subjects, in accordance with the data found in other publications. These scores were also higher than those reported by the single amputee patient. Regarding the QoL, there’s more observed variation, both in-domain and between domains, which is consistent with other studies' findings, where the main differences in scoring are seen in specific domains of the questionnaire, rather than in the final score between the surgery groups.

Our study has several limitations. The sample size, particularly for functional and QoL assessments, was small, and the disparity in group sizes limited definitive comparisons between surgical modalities. Selection bias, stemming from the medical criteria guiding surgical decisions, further skewed survival rates. Additionally, combining participants from two distinct healthcare environments (Portugal and PSAC countries) introduced heterogeneity, complicating comparisons with studies focused on single demographics.

## Conclusions

This study provides valuable insights into the clinical and surgical management of pediatric appendicular bone sarcomas, with a particular focus on survival, functional outcomes, and QoL. While LSS consistently demonstrated superior functional preservation, QoL outcomes were excellent in the amputation group, although based on limited responses. However, significant disparities in survival rates were observed in resource-limited settings. Children from PSAC presented with more advanced disease stages and poorer outcomes, highlighting the critical role of timely diagnosis and equitable access to healthcare.

The findings reinforce the importance of early intervention, as advanced disease stages characterized by metastasis or larger tumor sizes remain strong predictors of poor prognosis. Although AMP was associated with worse outcomes, this reflects the severity of the disease at the time of surgical decision-making rather than the procedure itself.

This study also highlights the need for larger, multicentric, and globally inclusive research efforts to better understand and address disparities in treatment outcomes for pediatric bone sarcomas. Improving healthcare infrastructure and access in resource-limited regions is essential to achieving equitable care and improving survival rates for this vulnerable population. Ultimately, the goal should not only be to extend survival but also to ensure that children retain their function and QoL after treatment.
